# Privacy Law Protection Based on the Information Security Assurance Algorithm

**DOI:** 10.1155/2022/8006605

**Published:** 2022-09-09

**Authors:** Zhanjiang Wang, Qifeng Yue

**Affiliations:** School of Law, Shanghai University of Finance and Economics, Shanghai 200433, China

## Abstract

With the continuous development of network technology, the production and lifestyle of human beings are also quietly changing. People find that the network makes life faster and more convenient and also makes our life more “transparent.” The openness, sharing, and convenience of the network make it easier to infringe on the privacy rights of others, bringing an unprecedented impact on traditional privacy protection. This paper studied information security assurance-related algorithms and built an information security assurance model. Through this model, some privacy violation events existing in the network today were analyzed. Then, a corresponding questionnaire was constructed according to some of the problems of these events. The questionnaire is mainly used for research on people of all age groups, mainly to study their understanding of relevant legal knowledge and their views on the law. A total of 188 valid questionnaires were collected in this survey. Only 37.23% people knew a lot about the law. Most of them learned it from the Internet, and they rarely learned about it from relevant books. About 70% people believe that the law plays a big role in privacy protection, and they will choose legal help if they encounter privacy violations. However, this is inconsistent with some data obtained from the information security assurance model. Through analysis, it can be seen that although they want legal help, they lack the relevant knowledge of the law, and they do not know whether the privacy of individuals has been violated. Therefore, it is necessary to strengthen personal knowledge of privacy protection laws and improve the personal information legal protection and fair use system.

## 1. Introduction

Infringement of personal information is concealed, and information users are good at covering up their own behavior with the help of various information technologies, which make it difficult to define and obtain evidence of their behavior. At the same time, the absence of relevant legal systems, insufficient government supervision, and vague industry rules make it difficult to achieve effective legal protection. On the other hand, the lack of protection leads to abuse, and excessive protection pressure will hinder the development of the personal information industry. The inherent conflict between protection and use makes this a tricky one. The invasion of personal privacy in the Internet age has become a hidden worry and trouble for many netizens, and the security of Internet privacy has become a difficult problem. It can be said that the protection and use of personal information is a relatively new problem in the world. Many experts, scholars, and staff from various countries and regions are struggling to be on the front line of solving this problem. Therefore, how to successfully deal with information security issues to ensure the security of development is of great practical significance to many countries. The more common or deadly information security issues become, the more critical it is to have effective security assurance.

In order to protect the privacy of individuals, researchers have conducted research on the legal aspects of privacy protection. Among them, Mohamed and Chen [1] examined the potential relationship between the existence of a national data protection framework and its impact on e-health. They researched how college students share and use personal information through online media. The findings will be used to discuss and suggest improvements needed for the current iteration of Malaysia's data privacy laws [1]. Zapadka [2] studied the selected aspects of statistical secrecy, determining the extent to which the data collection and storage processes provided by the Public Statistics Act constitute justified legal interference with the constitutional rights and freedoms of natural people [2]. Danilovic [3] discussed Serbian employees' right to privacy at work in the early twenty-first century and its relationship to international regulations and practices. He proposed the establishment of an internal privacy policy with full respect for data protection regulations, which can provide a feasible and reasonable expectation of privacy at work [3]. Baruh et al. [4] investigated privacy concerns and literacy as predictors of online service and social networking site usage, information sharing, and adoption of privacy-preserving measures. However, their method has not been practically applied.

In order to further strengthen the protection of personal privacy, scholars have begun to study information security algorithms to analyze the network. Among them, Beebeejaun [5] studied the challenges posed by current laws, which are based on assessing the legal framework for the rapid growth of fintech [5]. Dan [6] examined the role of information assurance in managing data security by analyzing the formulation and introduction of the General Data Protection Regulation in May 2018 [6]. Saranya et al. [7] analyzed different types of cloud security issues and used a variety of information security assurance algorithms to generate keys and signatures to provide efficient security [7]. Nabi et al. [8] studied the application logic-related component risk, representing “the process of unifying security assurance properties” to deal with logic vulnerabilities in the system [8]. Alexander [9] investigated whether the AHP model can be effectively applied to the prioritization of information assurance defense-in-depth measures. Findings showed that AHP does not affect the relationship between information technology analysts' prioritization of the five defense-depth dependent variables and independent variables such as cost, ease of use, and effectiveness in protecting an organization's devices from attack [9]. However, the practicality of their algorithm is not great.

This paper established a model of an information security assurance system by studying information security assurance algorithms. Using this model to analyze the current network and then combining it with the questionnaire to study a large number of people, it is found that there are a large number of privacy violations on the network. However, these groups have a lack of legal knowledge about privacy protection, which further exacerbates the lack of privacy. Therefore, new strategies need to be developed to protect personal privacy. The innovation of this paper is as follows: based on the original information security assurance algorithm, it built a more comprehensive information security assurance system model and optimized the algorithm of the model.

## 2. Information Security Assurance Algorithms and Privacy Protection

### 2.1. Information Security

**Figure 1:**
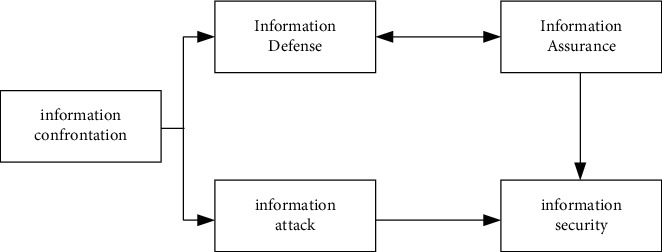
Relationship diagram of information security, information assurance, and information confrontation.

The concept of information security has been continuously expanded and deepened with the development of information technology. Initial information security mainly refers to communication security. The current concept of information security refers to information security assurance that comprehensively protects information infrastructure, application services, and information content [10, 11]. The connotation of information security has been extended to the confidentiality, integrity, authenticity, controllability of information, availability of information infrastructure, and nonrepudiation of interactive behavior, and it also includes active defense in information operation conditions. The relationship between information security, information assurance, and information confrontation is shown in Figure 1.

The research content of information security includes two themes: data security and network security. Closely related to this research topic are the security and reliability of computer mainframes and network environments. There are various measures taken for the security and reliability of the computer host and network environment, such as virus prevention, network firewall, and access control [12].

Data security means that the user's data information is not destroyed, the read and write can be consistent, and unauthorized access can be rejected. It is the most fundamental research content of information security. There are many measures to ensure data security, including physical measures, such as strict control of important computer rooms, strict management of hosts, and data backup. It also includes technical measures, such as data consistency check, data redundancy, and network security. In the network environment, network security is an important fundamental measure to ensure data security.

Network security refers to the use of network management control methods and technical measures to ensure the confidentiality, integrity, and availability of data and information in the network environment [13]. The main goal of network security is to ensure that information transmitted over the network and data on network hosts are not added, altered, lost, or illegally read, and host systems in the network are not illegally exploited. These levels are divided into physical security, host system security, and network security detection.

**Figure 2:**
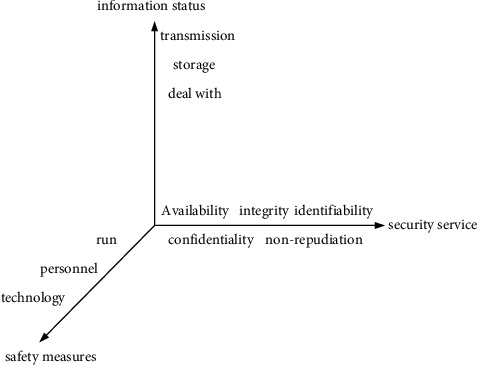
Time section of the information assurance model.

The information security model is an important basis for describing and characterizing the system and information security features, and it is also the foundation of security thinking and technology. The highest-level security system requires formal design and correctness proof [14]. Information assurance is an interdisciplinary subject involving many fields, including not only information security technology but also security management technology, the technical framework of security protection, and the life cycle process of information assurance.  Figure 2 shows the “section” of the extended McCumber model in the time dimension. The multidimensional model fully describes the various elements contained in information assurance in detail and also reflects the connections and relationships between various elements. The four dimensions included in the information assurance model are information states, security services, security countermeasures, and time.

**Figure 3:**
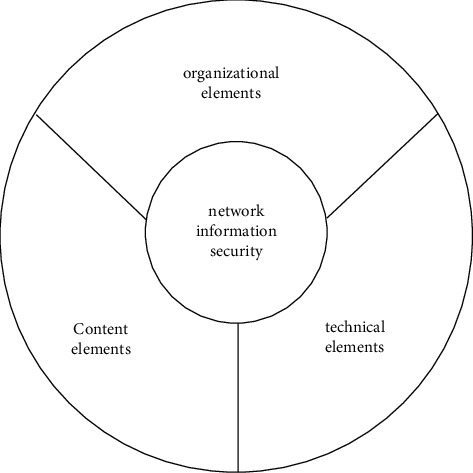
Basic elements of the network information security system.

In order to realize the security of network information, users should establish the capabilities of information collection, protection, and coordination in the information domain. Then, based on this ability, they put themselves in a more favorable position relative to the attacker's information. In this way, the ability to establish, generate, and share high-quality attitude perceptions, jointly understand defense intentions, and self-synchronization of defense processes in the cognitive domain is further realized. As a state of network information security, the network should have the following three specific capabilities: (1) immunity to external damage; (2) to ensure the normal and effective performance of its functions and to maintain its openness and scalability; (3) to protect its integrity and stability [15]. Network information security should include three elements, as shown in Figure 3.

**Figure 4:**
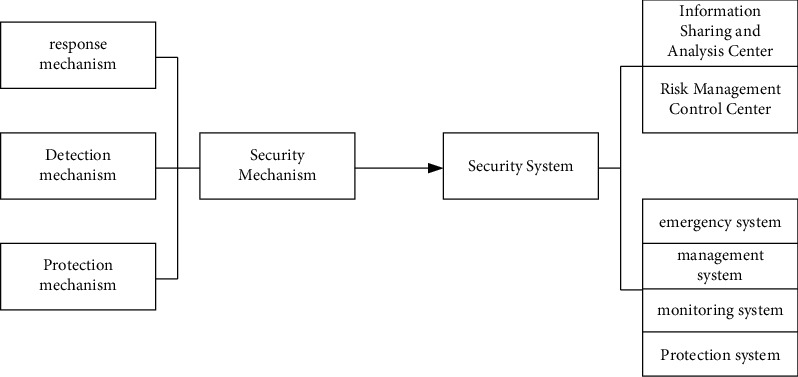
Composition model of the network information security assurance system.

Based on the above analysis, it can be seen that an effective and complete network information security system should have three mechanisms: protection, detection, and response. Based on this, a complete network information security assurance system should include four systems: protection capability, monitoring capability, management mechanism, and emergency mechanism, as shown in Figure 4.

**Figure 5:**
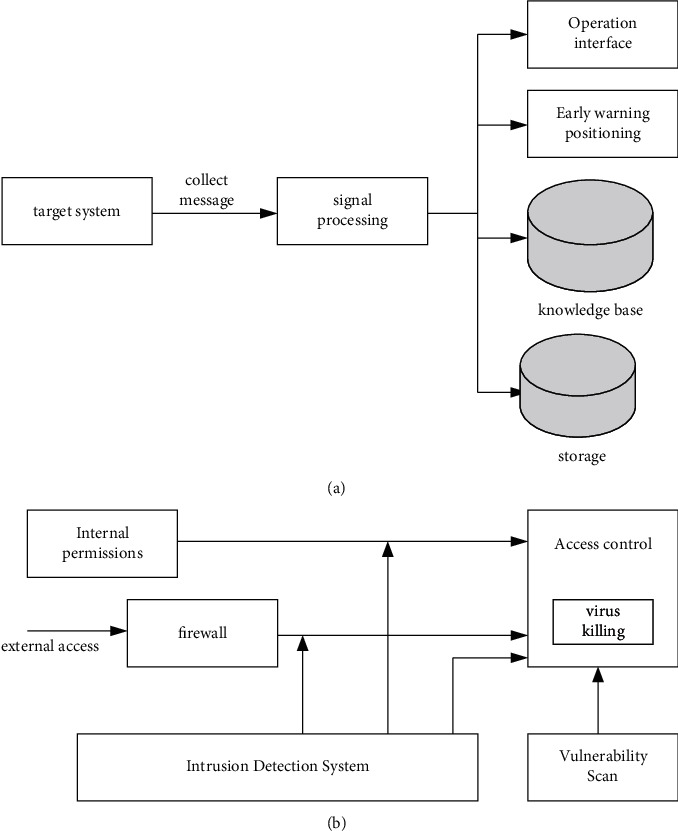
Intrusion Detection. (a) Principle of the intrusion detection system. (b) The relationship between intrusion detection and other security technologies.

An intrusion is any attempt to compromise the integrity, confidentiality, and availability of computer system resources or to override the security mechanisms of a computer or network. Intrusion behaviors include the following two aspects: (1) maliciously obtaining system control rights beyond the legal scope; (2) behaviors that cause certain harm to the network system through system vulnerabilities. Intrusion detection is a technique used to detect intrusions in computer networks that violate security policies [16]. By collecting and analyzing key information in the network and host systems in real time, it timely detects and reports the abuse of resources by illegal users and legitimate users in the system and makes appropriate responses. The schematic diagram of intrusion detection and its relationship with other technologies are shown in Figure 5.

From Figure 5(a), it can be seen that the intrusion detection is placed behind the firewall, and its monitoring or information processing of the network generally does not affect the performance of the system. It provides real-time protection against internal and external attacks and more. It can be seen from Figure 5(b) that in the overall security system of a network, intrusion detection systems, firewalls, vulnerability scanning, and antivirus software work together to describe the role of IDS in the network security system [17, 18]. The main role and function of IDS should be reflected in the following points: (1) subjecting the activities of users and systems to its monitoring and analysis; (2) configuration and weaknesses of audit systems; (3) assessing the integrity of critical system data files.

At present, the most widely used intrusion detection systems are mainly expert systems and statistical analysis techniques. The expert system is currently the most widely used technology in abuse detection systems. It uses domain-expert knowledge and reasoning mechanisms and has certain intelligence. It is mainly used for the abuse detection system. The main method of this technology is to set the domain expert knowledge as an intrusion detection rule and judge the legitimacy of the behavior by detecting whether the user's behavior is consistent with the detection rule.

**Figure 6:**
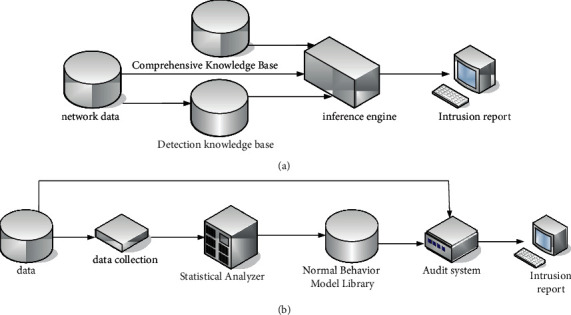
Intrusion detection system. (a) The schematic diagram of expert system intrusion detection. (b) Principle of the intrusion detection system based on statistical analysis.

A statistical survey is one of the earliest technologies used in intrusion detection technology, and many commercial versions of intrusion detection products still use this technology [19]. The system assumes that the user's behavioral habits are regular and then builds an auditor of the system by analyzing and modelling the user's historical behavior or collecting early evidence of the system. The auditor judges the intrusion by checking whether the user's behavior conforms to the normal behavior model. Their schematics are shown in Figure 6.

Figure 6(a) shows the schematic diagram of the intrusion detection of the expert system. It can be seen from the figure that the key technologies in the expert system are as follows: (1) The detection knowledge base stores the knowledge of intrusion detection rules. It is operated by experts in the security field and can add detection rules at any time. (2) The inference engine simulates the reasoning ability of the human brain and judges whether the network behavior is legal or not according to the intrusion rules and the rules of the comprehensive knowledge base. (3) The comprehensive knowledge base stores data of various networks and hosts, intermediate inference results, and final results. The system has a high detection efficiency for known intrusion behaviors but does not have the ability to detect unknown intrusion behaviors. The acquisition of intrusion detection rules requires human intervention, and the ability to automatically acquire knowledge is low.

Figure 6(b) shows the schematic diagram of the intrusion detection system based on statistical analysis. It can be seen from the figure that the detection method of the detection system uses some statistical variables to describe the behavior of users or systems. The main functions of the system include selecting appropriate statistics, statistical data analysis and intrusion judgment, and adaptive update of statistical models. Intrusion detection systems based on statistical analysis have a certain learning ability and adjust the model data of the auditor by continuously analyzing the historical behavior of users or systems. However, it also has a fatal disadvantage that illegal users can train intrusion detectors through a large number of abnormal behaviors to achieve “legitimate” purposes. In addition, the real-time performance of the system is poor.

### 2.2. Information Security Assurance Algorithm

**Figure 7:**
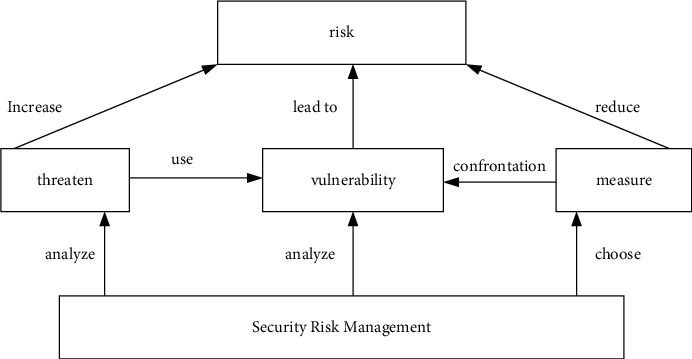
Information security assurance model based on risk management.

By means of information security assurance, the risk of information and information systems can be reduced. Considering the differences in the degree of informatization between different countries and the gap in information technology, this paper puts forward the idea of building an information security assurance system based on risk management, highlighting the importance of risk management in an information security system. The information security assurance model is shown in Figure 7.

Through the risk analysis, the security threats existing in the information environment and the vulnerability analysis of the system are given. According to the context of information security assurance and the system vulnerability obtained by the risk analysis, appropriate security control measures are selected. At the same time, the cost of safeguard measures and the value of information assets are analyzed in order to obtain the optimal risk management scheme [20].

The BP neural network is the most widely used network at present, which is usually composed of an input layer, several hidden layers, and an output layer. The BP algorithm is a supervised learning algorithm. Its main idea is to use gradient search technology for known learning sample pairs, in order to minimize the mean square error between the actual output value of the network and the expected output value. If the expected output cannot be obtained in the output layer, it is transferred to backpropagation, and the error of the output signal is returned along the original connection path. The weight of each layer of neurons is modified to minimize the error. The basic evaluation process is as follows:

Network state initialization: assigning a random number between the connection weight *w*_*ij*_, *v*_*jt*_ and the threshold *θ*_*j*_, *γ*_*t*_ endow and(−1,1).

Entering the first learning sample pair.

Calculating the input *u*_*j*_ and output *h*_*j*_ of each neuron in the middle layer, namely,

(1) $$\begin{array}{c} u_{j}=\overset{n}{\underset{i=1}{\sum }}w_{ij}x_{i}-\theta_{j}\text{,}\\ h_{j}=f\left(u_{j}\right)\text{.} \end{array}$$

Among them, *f* adopts the sigmoid function, i.e.,

(2) $$\begin{array}{c} f\left(u\right)=\frac{1}{\exp\;\left(-u\right)+1}\text{.} \end{array}$$

Calculating the input *l*_*t*_ and output *y*_*t*_ of each neuron in the output layer, namely,

(3) $$\begin{array}{c} l_{t}=\sum v_{jt}h_{j}-\gamma_{t}\text{,}\\ y_{t}=f\left(l_{t}\right)=\frac{1}{\exp\left(l_{t}\right)+1}\text{.} \end{array}$$

Calculating the weight error *δ*_*t*_ connected to the output layer unit *t*, which is as follows:

(4) $$\begin{array}{c} \delta_{t}=\left(c_{t}-y_{t}\right)y_{t}\left(1-y_{t}\right)\text{.} \end{array}$$

Among them, *c*_*t*_ in the above formula is the expected value of the sample.

Calculating the weight error *σ*_*t*_ connected to the intermediate layer unit j, as shown in the following equation:

(5) $$\begin{array}{c} \sigma_{t}=\overset{q}{\underset{t=1}{\sum }}\delta_{t}v_{jt}h_{j}\left(1-h_{j}\right)\text{.} \end{array}$$

Updating the connection weight *v*_*jt*_ and the threshold *γ*_*t*_, i.e.,

(6) $$\begin{array}{c} v_{jt}\left(N+1\right)=v_{jt}\left(N\right)+\alpha \sigma_{t}h_{j}\text{,}\\ \gamma_{t}\left(N+1\right)=\gamma_{t}\left(N\right)-\beta \delta_{t}\text{.} \end{array}$$

Updating the connection weight *w*_*ij*_ and the threshold *θ*_*j*_, i.e.,

(7) $$\begin{array}{c} w_{ij}\left(N+1\right)=w_{ij}\left(N\right)+\alpha \sigma_{j}x_{i}\text{,}\\ \theta_{j}\left(N+1\right)=\theta_{j}\left(N\right)-\beta \sigma_{j}\text{.} \end{array}$$

Enter the next learning sample pair and return to step 3 until all *z* pattern pairs are trained.

Starting a new round of learning and training until the following conditions are met:

(8) $$\begin{array}{c} \left|\overset{z}{\underset{k=1}{\sum }}E_{k}\right|\leq \varepsilon \text{.} \end{array}$$

Among them, *ε* is the preset accuracy and *E*_*k*_ is the mean square error, i.e.,

(9) $$\begin{array}{c} E_{k}=\frac{1}{2}{\overset{q}{\underset{t=1}{\sum }}\left(c_{t}-y_{t}\right)}^{2}\text{.} \end{array}$$

There are more than 100 original data features in the network, which belong to multifeature, high-noise, and nonlinear datasets. If such high-dimensional data are directly used for classification, the computational load of the classifier will be very large. In particular, there will also be a large number of features irrelevant to data classification in the features, which are more likely to reduce the classification accuracy of the classifier. Therefore, before data classification, it is necessary to perform feature selection on the original network data, that is, data dimensionality reduction. The features that are most conducive to data classification are selected from them, thereby reducing the computational complexity of the classifier and improving the accuracy of classification.

The essence of feature selection is a process of optimizing computation, and pattern recognition is still one of the typical solutions. That is to say, it is necessary to find a criterion and express it with a formula, so that the formula reaches the extreme value in the calculation process [21]. In the process of feature evaluation, the evaluation methods are roughly divided into two categories. One is the distance-based separability criterion, that is, the criterion is based on calculating the dispersion degree of the sample in the feature space; the other is based on the probability density distribution as the basis for judgment.

Supposing the feature candidate set *X*=[*x*_1_, ..., *x*_*n*_], in its defined n-dimensional feature space, *d*=(*x*_*ik*_, *x*_*jt*_) is used to represent the distance metric between the *k-*th sample in the *i*-th class and the *l*-th sample in the *j-*th class. Then, the distance metric value can be defined as per the Euclidean formula and other calculations. Considering the computational complexity of the formula, it is converted into the corresponding overall matrix to measure and calculate, and the overall distribution matrix calculation formula is as follows:

The *i*-th mean vector can be expressed as follows:

(10) $$\begin{array}{c} {\bar{X}}^{w_{i}}=\frac{1}{N}\underset{x\in w_{i}}{\sum }X\text{.} \end{array}$$

The mean vector of the sample aggregate can be expressed as follows:

(11) $$\begin{array}{c} \bar{X}=\frac{1}{N}\overset{M}{\underset{i=1}{\sum }}p\left(w_{i}\right){\bar{X}}^{w_{i}}\text{.} \end{array}$$

Type *i* covariance can be expressed as follows:

(12) $$\begin{array}{c} \sum_{i}=\frac{1}{N_{i}-1}\underset{X\in w_{i}}{\sum }\left(X-{\bar{X}}^{w_{i}}\right){\left(X-{\bar{X}}^{w_{i}}\right)}^{T}\text{.} \end{array}$$

The total covariance of the sample can be expressed as follows:

(13) $$\begin{array}{c} \sum =\frac{1}{N-1}\sum \left(X-\bar{X}\right){\left(X-\bar{X}\right)}^{T}\text{.} \end{array}$$

The intraclass scatter matrix for the *i*-th class can be expressed as follows:

(14) $$\begin{array}{c} S_{i}=E\left|\left(X-{\bar{X}}^{w_{i}}\right){\left(X-{\bar{X}}^{w_{i}}\right)}^{T}\right|\text{.} \end{array}$$

The population within the class scatter matrix can be expressed as follows:

(15) $$\begin{array}{c} S_{w}=\overset{M}{\underset{i=1}{\sum }}p\left(w_{i}\right)S_{i}\text{.} \end{array}$$

The population between the class scatter matrix can be expressed as follows:

(16) $$\begin{array}{c} S_{B}=\overset{M}{\underset{i=1}{\sum }}p\left(w_{i}\right)\left({\bar{X}}^{w_{i}}-\bar{X}\right){\left({\bar{X}}^{w_{i}}-\bar{X}\right)}^{T}\text{.} \end{array}$$

The population scatter matrix can be expressed as follows:

(17) $$\begin{array}{c} S_{T}=E\left|\left(X-\bar{X}\right){\left(X-\bar{X}\right)}^{T}\right|\text{.} \end{array}$$

*S*_*T*_ and *S*_*B*_, *S*_*w*_ have the following relationship:

(18) $$\begin{array}{c} S_{T}=\left(S_{B}+S_{w}\right)\text{.} \end{array}$$

The intraclass scatter matrix represents the scatter of each sample point around its mean. The interclass scatter feature represents the distance distribution between classes, which depends on the sample category attributes and division. The overall scatter matrix has nothing to do with the sample division and attributes.

### 2.3. Privacy Protection

The essence of privacy mainly includes three parts, namely, private space, information, and activities that are not disturbed by the outside world. It also has the premise that is harmless to society and others. The privacy right is the right of citizens to make their own decisions about their personal life and information and has nothing to do with the public interest.

Privacy protection is mainly performed through the method of government legislation to formulate relevant legal regulations. The basic principles and specific systems for the protection of online privacy rights are legally established, and corresponding judicial or administrative remedies are established on this basis. The use of information makes the traditional closed society transition to transparent society. Personal information should not be locked in a “coffer” but should be put into the “big warehouse” of society to give full play to its value. The legal protection of personal information should not only protect individuals but also affirm the legality of the use of personal information by information users such as governments and commercial organizations. The purpose of legal protection is to seek coordination between the development and utilization of information resources and the protection of personality rights to reasonable use of personal information.

## 3. Experiments of Privacy Protection Law

### 3.1. Questionnaire of Privacy Protection Laws

This questionnaire set up a survey on different groups of people on privacy protection laws and conducted a survey on people of different age groups, as shown in Table 1. A total of 200 questionnaires were distributed in this survey, and 188 valid questionnaires were recovered, with a recovery rate of 94%.

It can be seen from Table 1 that in this questionnaire survey on privacy protection legal issues, the majority of people gave positive responses. Among them, 54.26% were teenagers aged 18–25, which were mainly students, followed by workers aged 25–40, accounting for 28.19% of the total questionnaires. The rest of the questionnaires were mainly attributed to minors under the age of 18 and people over 40. Because they mainly had little contact with the Internet, the ratio is relatively small, accounting for 9.57% and 7.98%, respectively.

In response to the concept of privacy and privacy rights, this questionnaire surveyed and found that they mainly understood privacy and privacy rights in a relatively simple way. They simply believed that it was their own personal information, and privacy rights also stayed in personal information. Only a few people have a comprehensive understanding of privacy and privacy rights, and the survey results are shown in Table 2.

From Table 2, it can be seen that only 57 people have learned about privacy and privacy rights, and the other 131 people have not seriously understood privacy and privacy rights. Thirty of the 53 people aged 25 to 40 have seriously understood it. It shows that more than half of people in this age group will learn about privacy protection laws, which are closely related to their work experience. It makes them more concerned about their personal information. Only 21 out of 102 aged 18 to 25have learned about privacy and privacy rights, which may also include students who majored in law. It shows that contemporary teenagers do not pay much attention to privacy concerns, thinking that these have nothing to do with themselves. However, in fact, privacy and privacy rights are closely related to our daily lives. As for minors, they are less concerned about privacy and privacy rights.

**Figure 8:**
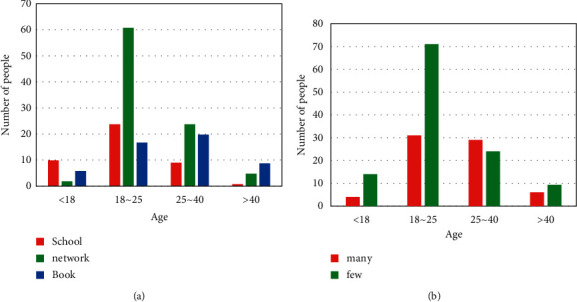
Knowledge of privacy protection laws. (a) Access to privacy shield laws. (b) Knowledge of privacy shield laws.

In order to further understand people's understanding of privacy protection laws, this questionnaire also raised a number of legal questions. Among them, about the two questions “How did you learn about privacy protection laws?” and “Do you know more or less about privacy laws?,” the received questionnaires have been organized, and the questionnaire data are shown in Figure 8.

From Figure 8(a), it can be seen that people in the age group of 18–40 mainly learn about privacy protection laws through the Internet, while those who are underage mainly learn about privacy protection laws through schools and books. For people over 40 years old, because they have little contact with schools and little use of the Internet, their information about privacy protection laws mainly comes from books. For one of them from the school, it may be because he is a school teacher. In Figure 8(b), it can be seen that there are significantly more people who know little about privacy protection laws than those who know a lot about privacy protection laws. This shows that contemporary people do not have a clear understanding of the importance of privacy protection laws, and most people have the mentality that privacy violations will not fall on their heads. However, privacy violations happen to us all the time.

**Figure 9:**
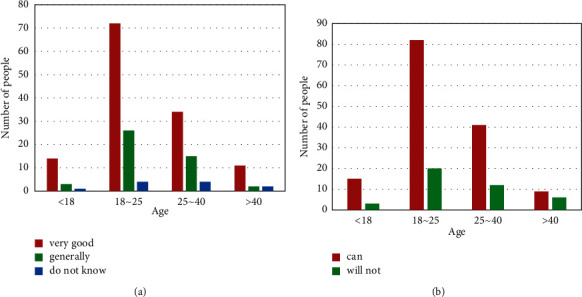
Views on privacy protection laws. (a) How effective the law is for privacy protection. (b) Whether they will seek legal aid.

In order to understand contemporary views of today's privacy protection laws, this questionnaire asked the two questions “How effective do you think the laws are in protecting privacy today?” and “Would you seek legal aid if your privacy was violated?.” The data are shown in Figure 9.

It can be seen from Figure 9(a) that the vast majority of people agree with the current law and believe that the law can protect privacy. A large part of the population will choose to seek legal protection, but some people do not choose to seek legal help, as shown in Figure 9. Although there are not many people, it further illustrates that the current privacy protection laws may not fully protect privacy. There are still some problems, which make some people not want to seek legal help.

**Figure 10:**
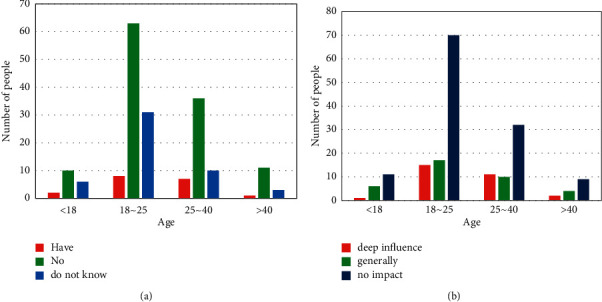
Reasons for lack of legal knowledge. (a) Whether there is any publicity about law. (b) Degree of influence of legal knowledge on daily life.

In addition, this questionnaire also conducted a questionnaire survey on the lack of legal knowledge among the population. The questionnaire questions were as follows: “Do you have legal popularization in your area?” and “Do you think the impact of legal knowledge on daily life?.” The questionnaire data are shown in Figure 10.

From Figure 10(a), it can be seen that most of the people in the area have no legal publicity, which leads them to not understand legal knowledge, especially some people aged 18–40. From Figure 10(b), most people think that legal knowledge has no effect on daily life, while a very small number of people think that legal knowledge has a deep impact on daily life, especially people aged 25–40. Most of this group of people have married and started a business, so they will be more concerned about the daily life of the family.

In order to find out whether people are interested in legal knowledge, the questionnaire was surveyed on legal videos. The question was “What would you do if you swiped a short video about the law?,” and the data from all the questionnaires were analyzed, and the results are shown in Table 3.

As can be seen from Table 3, the number of people who will watch short videos related to the law is not very large, and most of them will watch for a few seconds or skip them directly. Especially for teenagers in the age group of 18–25, the ratio of being able to watch a complete short video about the law is even less. 28 people will choose to watch it. This is because of the development of technology and the diversity of short videos. Many young people aged 18–25 pursue some funny and celebrity videos and other videos that are not useful in life, so they will choose to skip short legal videos when they see them. The same is true for other age groups, and the biggest aspect may be the different pursuit of short videos. However, about one-third of people over the age of 40 choose to watch a short video about the law when they swipe it, because this age group has a general awareness of how to make life better.

Combining all data analysis, most people now lack legal knowledge, especially regarding privacy protection laws. Although they know that laws are important, they do not have a detailed understanding of the nature of the relevant laws, which leads them to further ignore the importance of laws. As a result, when their privacy may be violated, they will not choose the law to protect themselves.

### 3.2. Suggestion

The first point is to improve the legal system. The first is to confirm the right to personal information and to define the subject and object of protection. The struggle for division makes the objects of legal protection have the right to maintain. Second, it is recommended that we distinguish personal information into private information and general information. The unreasonable use of private information is far more harmful to its subjects than that of general information. The protection of the former should be strengthened by the law, and the constraints on the use of the latter should be weakened.

The second point is to build a government supervision system to implement the legal protection of personal information and guide users to use it reasonably. Specifically, through the use of the licensing system, the entry threshold for the personal information industry is raised, and those commercial institutions that do not have the ability to use security measures and regulatory systems are excluded, and the work of the regulatory agency is focused. At the same time, the reward and punishment measures are clearly stipulated to play an exemplary role. Rewards are used to subsidize the efforts of enterprises in the process of reasonable use, whereas punishment is used to prevent abuse of illegal profits.

The third point is to respond to the legal protection of personal information from the perspective of industry autonomy. Through self-management, self-requirement, and self-promotion in the form of industry associations and industry codes, they should do a good job in the self-discipline of the industry and also participate in the balance of protection and use.

The fourth point is from an individual point of view. It is suggested that we improve the liability system and introduce a representative litigation model. The unequal proof capacity and response level should be balanced between information rights and information users to reduce the cost of the former rights of the protection process and increase the amount of damages so as to protect personal information, dispel the speculation of information users, and use personal information rationally.

## 4. Conclusion

This paper investigated and researched people of all age groups by means of questionnaires, mainly to explore the citizens' understanding of privacy protection laws and other laws. From all the data analysis of the questionnaire, it can be seen that people of all age groups have a serious lack of legal knowledge and little knowledge about privacy protection. As an intangible property, personal information plays an increasingly important role in the development of society. Therefore, it is necessary to strengthen citizens' knowledge of the law so that more citizens can use legal knowledge to protect themselves when they encounter privacy violations. People believe that with the gradual improvement of the country's legislation on privacy protection, the popularization of privacy protection education, and the protection of privacy rights, technological means will change with each passing day.

## Figures and Tables

**Table 1:** Survey distribution by age groups.

Age	Number of people	Proportion (%)
<18	18	9.57
18 ∼ 25	102	54.26
25 ∼ 40	53	28.19
>40	15	7.98

**Table 2:** Whether people know about privacy and privacy rights.

Age	Yes (number of people)	No (number of people)
<18	1	17
18 ∼ 25	21	81
25 ∼ 40	30	23
>40	5	10

**Table 3:** Practice of brushing short videos on legal aspects.

Age	See it all	Watch it for a few seconds	Skip straight through
<18	2	5	11
18 ∼ 25	28	36	38
25 ∼ 40	11	24	18
>40	5	4	6

## Data Availability

The datasets generated during and/or analyzed during the current study are not publicly available due to sensitivity and data use agreement.
